# Clinical and molecular characterization of Wilson's disease in China: identification of 14 novel mutations

**DOI:** 10.1186/1471-2350-12-6

**Published:** 2011-01-11

**Authors:** Xin-Hua Li, Yi Lu, Yun Ling, Qing-Chun Fu, Jie Xu, Guo-Qing Zang, Feng Zhou, Yu De-Min, Yue Han, Dong-Hua Zhang, Qi-Ming Gong, Zhi-Meng Lu, Xiao-Fei Kong, Jian-She Wang, Xin-Xin Zhang

**Affiliations:** 1 Department of Infectious Diseases, Ruijin Hospital, Shanghai Jiaotong University School of Medicine, Shanghai, China; 2 Sino-French Research Center for Life Sciences and Genomics, Ruijin Hospital, Shanghai Jiaotong University School of Medicine, Shanghai, China; 3 Institute of Infectious and Respiratory Diseases, Ruijin Hospital, Shanghai Jiaotong University School of Medicine, Shanghai, China; 4 The Center for Pediatric Liver Diseases, Children's Hospital of Fudan University; 5 Department of Pediatrics, Shanghai Medical College, Fudan University, Shanghai, China; 6 Liver Disease Research Center, Nanjing Military Command, Shanghai, China; 7 Department of Infectious Diseases, No. 3 People's Hospital, Shanghai Jiaotong University, School of Medicine, Shanghai China; 8 Department of Infectious Diseases, No. 6 People's Hospital, Shanghai Jiaotong University, School of Medicine, Shanghai, China

## Abstract

**Background:**

Wilson's disease (WND) is a rare autosomal recessive disorder. Here we have evaluated 62 WND cases (58 probands) from the Chinese Han population to expand our knowledge of *ATP7B *mutations and to more completely characterize WND in China.

**Methods:**

The coding and promoter regions of the *ATP7B *gene were analyzed by direct sequencing in 62 Chinese patients (58 probands) with WND (male, n = 37; female, n = 25; age range, 2 ~ 61 years old).

**Results:**

Neurologic manifestations were associated with older age at diagnosis (p < 0.0001) and longer diagnostic delay (p < 0.0001). Age at diagnosis was also correlated with urinary copper concentration (r = 0.58, p < 0.001). Forty different mutations, including 14 novel mutations, were identified in these patients. Common mutations included p.Arg778Leu (31.9%) and p.Pro992Leu (11.2%). Homozygous p.Arg778Leu and nonsense mutation/frameshift mutations were more often associated with primary hepatic manifestations (p = 0.0286 and p = 0.0383, respectively) and higher alanine transaminase levels at diagnosis (p = 0.0361 and p = 0.0047, respectively). Nonsense mutation/frameshift mutations were also associated with lower serum ceruloplasmin (p = 0.0065).

**Conclusions:**

We identified 14 novel mutations and found that the spectrum of mutations of *ATP7B *in China is quite distinct from that of Western countries. The mutation type plays a role in predicting clinical manifestations. Genetic testing is a valuable tool to detect WND in young children, especially in patients younger than 8 years old. Four exons (8, 12, 13, and 16) and two mutations (p.Arg778Leu, p.Pro992Leu) should be considered high priority for cost-effective testing in China.

## Background

Wilson's disease (WND; OMIM#277900) is a rare autosomal recessive disorder that is caused by abnormal copper metabolism; its prevalence is approximately 30 cases per million people [1-4]. The excessive copper accumulation in various organs, primarily the liver, brain, kidney, and cornea, results in a spectrum of hepatic and neurologic abnormalities [5-7]. Clinical presentation is highly heterogeneous [8,9]; patients can present with hepatic symptoms, neurologic symptoms, or both. The age of onset ranges from 2 to 70 years [10-12]. Diagnosis of WND is based on clinical symptoms (hepatic symptoms, neurologic symptoms, and cornea Kayser-Fleischer ring) and biochemical tests (elevated 24-h urinary copper, low plasma ceruloplasmin, and elevated liver copper concentrations) [1,2]. However, biochemical markers can be misleading [1,2], rendering WND diagnosis difficult in the absence of typical symptoms. For that reason, genetic testing has become the method of choice to establish a precise diagnosis [2,3].

The gene responsible for WND was first identified in 1993 and encodes a copper-transporting P-type ATPase (*ATP7B*; OMIM *606882) [13-15]. It is located on chromosome 13 (13q14.3-q21.1) and consists of 21 exons, which span a DNA region of approximately 100 kb. The *ATP7B *gene encodes 1465-amino acid membrane protein [13,15,16] that consists of six metal-binding domains, eight transmembrane segments, an ATP-binding domain typical of copper ATPases with a P-domain, an N-domain, and an A-domain with the TGE sequence motif [17-19]. ATP7B is a transporter in the copper secretory pathway that delivers copper for incorporation into apoceruloplasmin and excretion into the bile [6,20]. Impaired ATP7B function results in excessive cellular copper accumulation, thereby causing WND.

To date, more than 500 mutations have been identified in patients with WND, (Human Gene Mutation Database (Cardiff): http://www.hgmd.cf.ac.uk/ac/index.php and Wilson's Disease Mutation Database: http://www.wilsondisease.med.ualberta.ca/index.asp [21]). Most mutations are extremely rare and limited to individual patients. In studies of Caucasian populations, the p.His1069Gln mutation represents 37% to 63% of mutations [3,22]; however, the frequency and distribution of *ATP7B *mutations in Chinese WND patients has not been well studied. Only a few articles have reported *ATP7B *mutations in the Chinese population [23-31], and most of these studied only WND patients in the southern part of China. The present study therefore aimed to broaden the knowledge of *ATP7B *mutations in Chinese patients to determine whether genotype/phenotype correlations could be established.

## Methods

### Patients and control

A total of 62 Wilson's disease patients (58 probands) from 58 unrelated families were included from five medical centers in Shanghai, China (male, n = 37; female, n = 25; age range, 2.6 ~ 61 years old). Most of patients are children accounting for 80.6% (younger than 18 years old, n = 50). The clinical diagnosis of WND was based on a combination of clinical manifestations, laboratory tests and other features [1,2,32,33]. The main criteria used to establish the diagnosis of WND included: clinic manifestations, neurologic evaluation, corneal Kayser-Fleischer rings (K-F rings), liver function test, liver biopsy findings, liver copper concentration, serum ceruloplasmin concentration (0.2 g/L,enzymatic method), 24-h urinary copper concentration (upper limit of normal [ULN], 40 μg/24 hours) (Inductively Coupled Plasma Mass Spectrometry, America, Agilent) and *ATP7B *genetic testing. Based on the scoring system proposed at the 8th International Meeting on Wilson Disease in Leipzig, Germany in 2001 [32,34], the diagnosis of WND was established with a cumulative score of at least four.

Patients were classified according to clinical manifestations into the following groups: preclinical (presymptomatic; identified by family screening), hepatic manifestations (H1: acute hepatic WND; H2: chronic hepatic WND), neurologic manifestations (N1:associated with liver disease; N2: not associated with liver disease) [32,35].

Genomic DNA from 100 healthy Chinese individuals without WND was sequenced and analyzed to exclude the possibility that newly identified mutations were rare single nucleotide polymorphisms (SNPs).

Written informed consent was obtained from all patients or patients' legal guardians, and the study was approved by The Ethical Committee of of Shanghai Jiaotong University in accordance with the Helsinki Declaration.

### Genomic DNA extraction and mutation analysis

Genomic DNA was extracted from peripheral blood leukocytes with the Genomic DNA Purification Kit (Qiagen, Germany). Entire exons and their associated boundary regions were amplified by PCR with previously reported primers [36]. PCR products were analyzed using the Big-Dye Terminator Chemistry kit and the ABI377 automated DNA sequencer (Applied Biosystems, Foster City, CA, USA). The reference for cDNA sequence of *ATP7B *was submitted to GenBank (NM_000053.2). We used the standard nomenclature recommendations of the Human Genome Variation Society (HGVS) [37].

### Statistical analysis

Continuous variables (age, and ceruloplasmin, serum copper, and urinary copper concentrations) are expressed as number (n), mean and standard deviation (SD) or median and range. Continuous variables were checked the distribution for normality by "KS normality test" and "Shapiro-Wilk normality test". Continuous variables which were normally distributed were presented as mean and SD and were compared between groups by ANOVA test and *t *tests. Continuous variables which were not normally distributed in the analyzed population were presented as median and range and were compared between groups by Kruskal-Wallis ANOVA test and Mann-Whitney *U *test. Discrete variables (neurologic, hepatic, or presymptomatic phenotypes; presence or absence of K-F rings) were compared by χ^2 ^and Fisher's exact test, and expressed as percentages. Data were analyzed with SAS version 8.0. p < 0.05 was considered significant.

### Systematic literature review

In order to expand our knowledge of *ATP7B *mutation spectrum in Chinese population, we conducted a systematic review of the literature using the Wilson's Disease Mutation Database [21] and PubMed (finalized on July 1rst, 2010) with the combination of Wilson Disease and mutation(s) in the search field. We reviewed abstracts and retrieved articles that focused on the Chinese population. Our systematic review conformed to the guidelines laid out by PRISMA[38,39].

## Results

### Characterization of clinical phenotypes

We included 62 Chinese WND patients (58 probands) in the present study. Of these, 56 (90.3%) presented with liver disease, 4 (6.5%) were presymptomatic and received a diagnosis by familial screening, 19 (32.7%) presented with neurologic symptoms, and 25 (43.1%) presented with K-F rings at the slit-lamp examination. Presentations among the 56 patients with liver disease included liver cirrhosis (n = 31), abnormal biochemical liver function test results (n = 16), hepatic steatosis (n = 6), hepatomegaly (n = 3), liver failure (n = 2), and Coombs-negative hemolytic anemia (n = 1); other symptoms included fatigue, abdominal pain, edema, ascites, and jaundice. Presentations among the 19 patients with neurologic symptoms included deterioration in schoolwork (n = 5), encephalatrophy (detected by magnetic resonance imaging; n = 4), and trembling (n = 4); other symptoms included drooling, weak limbs, dysarthria, basal ganglia low-density lesions (detected by computed tomography), lack of motor coordination, myotonia, hearing loss, and inarticulacy. Median age at diagnosis 8.38 years (range 2.6 ~ 61), median age at symptom onset 7.56 years (range 2.5 ~ 60.75), median diagnostic delay was 4 months (range 2.6 ~ 61), median ceruloplasmin level was 7.35g/L (range 1 ~ 23.9), median 24-h urinary copper excretion was 222.5 μg per 24 h (rang 18 ~ 1727) (87.1% patients > 1 × ULN, 75.8% patients >2 × ULN), and mean serum ALT level was 130 ± 94.3 IU/L (66.7% patients > ULN, but 96% patients with abnormal liver function were younger than 8 years old [n = 27]).

**Figure 1:**
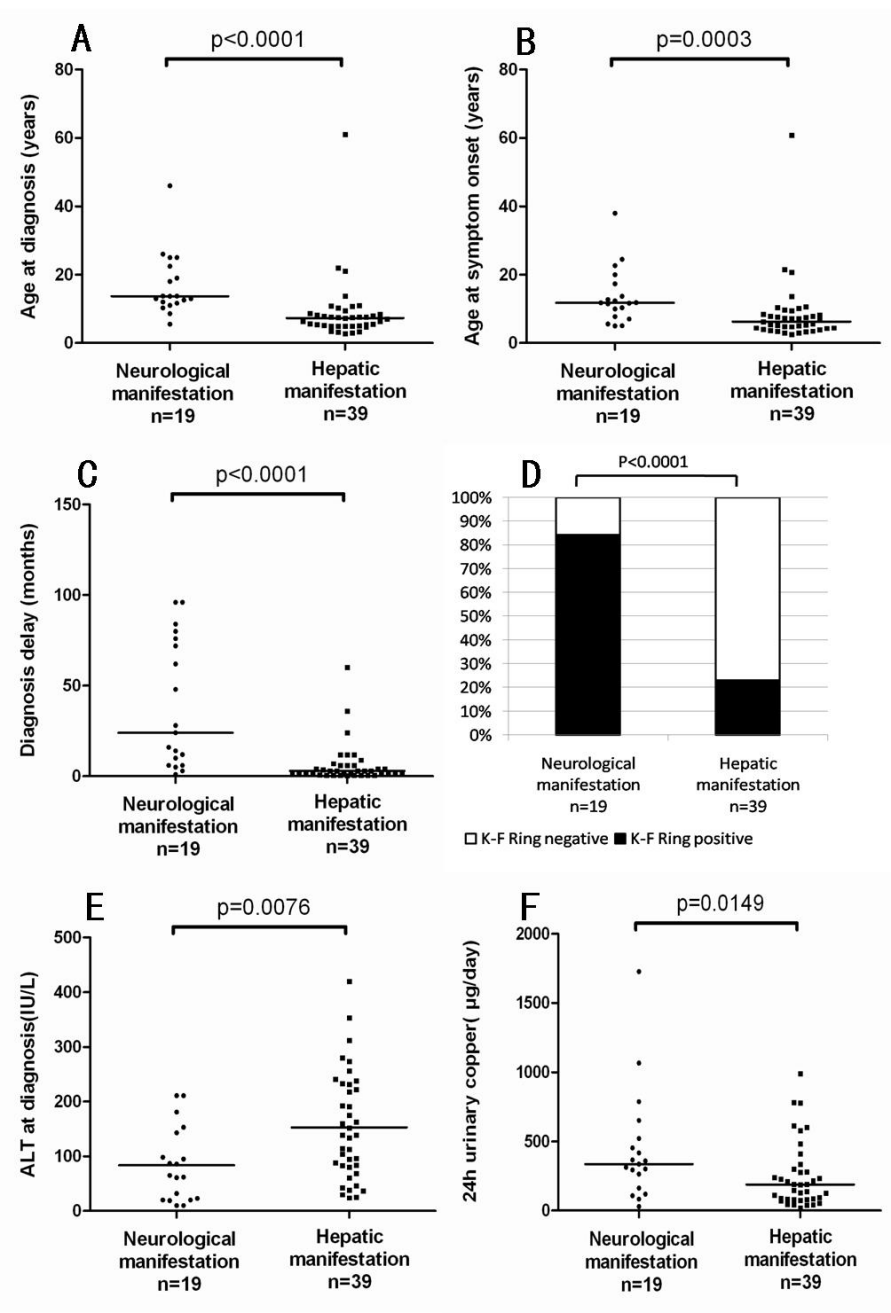
**Correlation of clinical manifestations with age at diagnosis (A), age at symptom onset (B), diagnostic delay (C), corneal Kayser-Fleischer rings (D), alanine transaminase levels at diagnosis (E) and 24-h urinary copper excretion at diagnosis (F) in Chinese patients with Wilson's disease**.

Compared with patients showing hepatic symptoms, patients with neurologic manifestations were significantly older at diagnosis (p < 0.0001; Figure 1A) and at symptom onset (p = 0.0003; Figure 1B), and had a longer diagnostic delay (p < 0.0001; Figure 1C), higher rate of K-F rings (p < 0.0001; Figure 1D), lower serum ALT levels (p = 0.0076; Figure 1E), and higher 24-h urinary copper levels (Figure 1F; p = 0.0149) (Additional file 1 : Supplementary TableS1).

**Figure 2:**
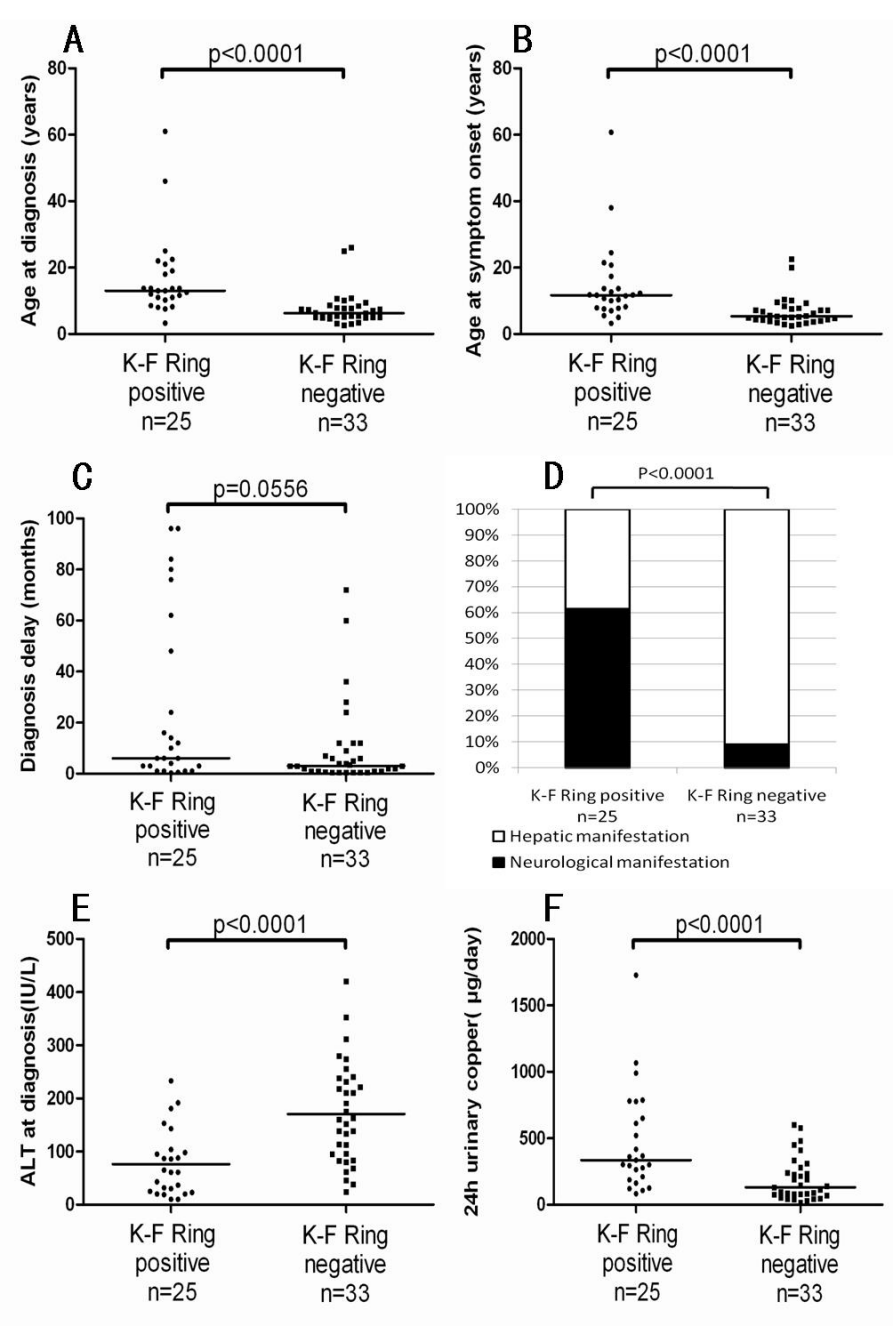
**Correlation of cornea Kayser-Fleischer (K-F) ring with age at diagnosis (A), age at symptom onset (B), diagnostic delay (C), corneal K-F Ring (D), ALT levels at diagnosis (E) and 24-h urinary copper levels at diagnosis (F) in Chinese patients with Wilson's disease**.

Because corneal K-F ring is an important clinical sign of WND, we compared patients positive for K-F rings with those who did not show this sign. Compared with K-F ring-negative patients, they were significantly older at diagnosis (p < 0.0001; Figure 2A) and older at symptom onset (p < 0.0001; Figure 2B), and had longer diagnostic delays tendency (p = 0.0556; Figure 2C), more neurologic manifestations (p < 0.0001; Figure 2D), lower serum ALT levels (p < 0.0001; Figure 2E), and higher 24-h urinary copper levels (p < 0.0001; Figure 2F) (Additional file 1 : Supplementary Table S2).

**Figure 3:**
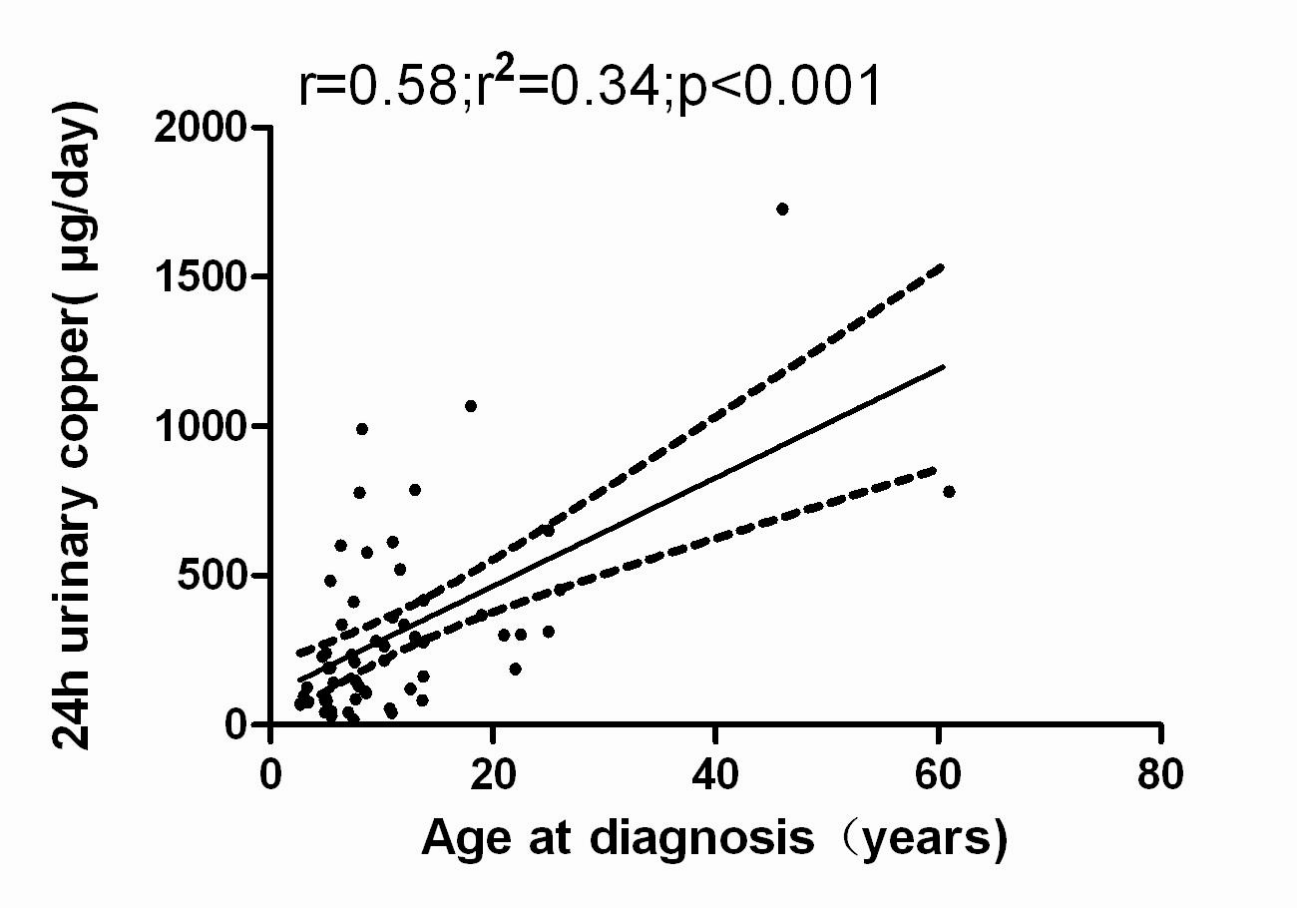
**Correlation between ages at diagnosis and 24-h urinary copper levels at diagnosis in Chinese patients with Wilson's disease**.

In addition, age at diagnosis was correlated with urinary copper concentration (r = 0.58, p < 0.001; Figure 3).

### Mutation analysis

**Figure 4:**
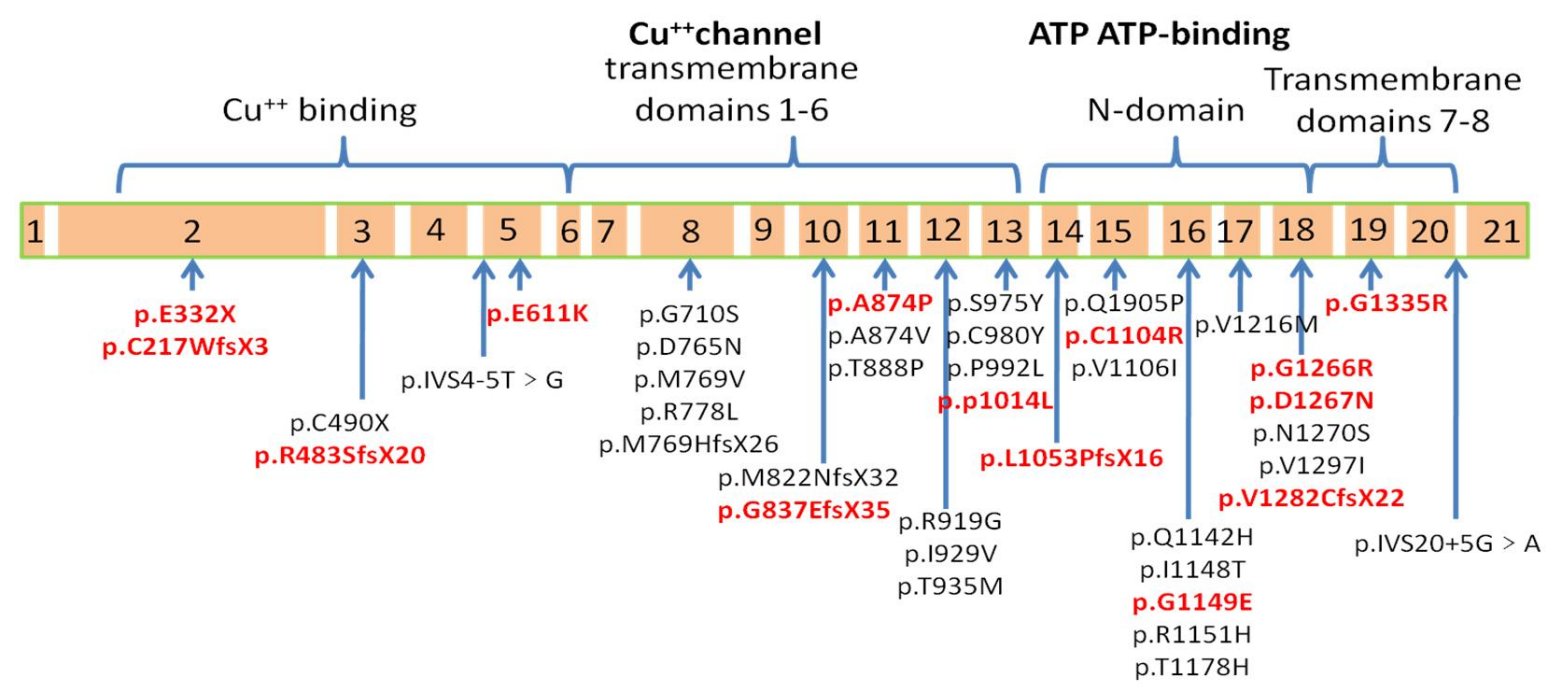
**Schematic representation of *ATP7B *mutations detected in the present study**. The novel mutations identified in this study are indicated in bold red letters.

By direct sequence analysis of the entire *ATP7B *gene coding and promoter regions, we identified 40 different mutations (missense mutations, n = 29; small deletion/insertions, n = 7; nonsense mutations, n = 2; splice-site mutations, n = 2) that accounted for 94.4% of the WND alleles in the 58 unrelated probands (Table 1). Of these 40 mutations, 14 were novel mutations that had not been reported previously. Further, these mutations were not found in the normal healthy Chinese participants (n = 100), excluding potential polymorphisms. These novel mutations include p.Glu611Lys, p.Ala874Pro, p.Pro1014Leu, p.Cys1104Arg, p.Gly1149Glu, p.Gly1266Arg, p.Asp1267Asn, p.Gly1335Arg, p.Glu332X, p.Cys271TrpfsX3, p.Arg483SerfsX20, p.Gly837GlyfsX17, p.Leu1053ProfsX16, p.Val1282CysfsX22 ( the sequence results are shown in Additional file 1 : Supplementary figure S1 and their location in the *ATP7B *gene exons is shown in Figure 4).

**Table 1:** ATP7B gene mutations in 58 Chinese WD probands

Nucleotide change	Nucleotide Sequence	Amino acid Change	exon	Gene region	No.of alleles	Alleles frequency(%)	Predicted Effect
Missense							
*c.1831 G > A	GAA > AAA	p.Glu611Lys	5	Cu6	1	0.86	Cu6
c.2128 G > A	GGT > AGT	p.Gly710Ser	8	TM2	1	0.86	Affects copper transport
c.2293 G > A	GAC > AAC	p.Asp765Asn	8	TM4	1	0.86	Disrupt cation channel&Tm4
c.2305 A > G	ATG > GTG	p.Met769Val	8	TM4	1	0.86	Affects copper transport
c.2333 G > T	CGG > CTG	p.Arg778Leu	8	TM4	37	31.9	Disrupt cation channel&Tm4
*c.2620 G > C	GCG > CCG	p.Ala874Pro	11	TM5	1	0.86	Affects copper transport
c.2621 C > T	GCG > GTG	p.Ala874Val	11	TM5	6	5.17	Affects copper transport
c.2662 A > C	ACC > CCC	p.Thr888pro	11	TM5	1	0.86	Affects copper transport
c.2755 C > G	CGG > GGG	p.Arg919Gly	12	TM5	2	1.72	Affects copper transport
c.2785 A > G	ATC > GTC	p.Ile929Val	12	TM5	1	0.86	Affects copper transport
c.2804 C > T	ACG > ATG	p.Thr935Met	12	TM5	1	0.86	Disrupt cation channel&Tm5
c.2924 C > A	TCC > TAC	p.Ser975Tyr	13	TM6	1	0.86	Disrupt cation channel&Tm6
c.2939 G > A	TGC > TAC	p.Cys980Tyr	13	TM6	1	0.86	Disrupt cation channel&Tm6
c.2975 C > T	CCC > CTC	p.Pro992Leu	13	TM6	13	11.2	Disrupt cation channel&Tm6
*c.3041 C > T	CCC > CTC	p.Pro1014Leu	13	TM6	1	0.86	Affects copper transport
c.3284 A > C	CAG > CCG	p.Gln1095Pro	15	ATP loop	1	0.86	Disrupts ATP binding
*c.3310 T > C	TGC > CGC	p.Cys1104Arg	15	ATP loop	1	0.86	Disrupts ATP binding
c.3316 G > A	GTC > ATC	p.Val1106ILe	15	ATP loop	2	1.72	Disrupts ATP binding
c.3426 G > C	CAG > CAC	p.Gln1142His	16	ATP loop	1	0.86	Disrupts ATP binding
c.3443 T > C	ATT > ACT	p.Ile1148Thr	16	ATP loop	1	0.86	Disrupts ATP binding
*c.3446 G > A	GGA > GAA	p.Gly1149Glu	16	ATP loop	1	0.86	Disrupts ATP binding
c.3452 G > A	CGT > CAT	p.Arg1151His	16	ATP loop	2	1.72	Disrupts ATP binding
c.3532 A > G	ACA > GCA	p.Thr1178Ala	16	ATP loop	1	0.86	Disrupts ATP binding
c.3646 G > A	GTG > ATG	p.Val1216Met	17	ATP bind	1	0.86	Disrupts ATP binding
*c.3796 G > C	GGG > CGG	p.Gly1266Arg	18	ATP loop	1	0.86	Disrupts ATP binding
*c.3799 G > A	GAT > AAT	p.Asp1267Asn	18	ATP hinge	1	0.86	Disrupt ATP hinge
c.3809 A > G	AAT > AGT	p.Asn1270Ser	18	ATP hinge	3	2.59	Disrupt ATP hinge
c.3889 G > A	GTC > ATC	p.Val1297Ile	18	ATP hinge	1	0.86	Disrupt ATP hinge
*c.4003 G > C	GGG > CGG	p.Gly1335Arg	19	TM7	2	1.72	Disrupt cation channel& Tm7
Nonsense							
*c.994 G > T	GAA > TAA	p.Glu332X	2	Cu4	3	2.59	Truncates protein
c.1470 C > A	TGC > TGA	p.Cys490X	3	Cu5	3	2.59	Truncates protein
Deletion							
*c.813delC	TTG**C**GTCT	p.Cys271TrpfsX3	2	Cu3	1	0.86	Frame shift/Truncates protein
*c.1448_1455del	CCA**GAGCAGTG**GCA	p.Arg483SerfsX20	3	Cu5	1	0.86	Frame shift/Truncates protein
*c.2510delG	GGG**G**AAA	p.Gly837GlufsX35	10	TM4	1	0.86	Frame shift/Truncates protein
Insertion							
c.2304dupC	CCC**C**ATG	p.Met769HisfsX26	8	TM4	4	3.45	Frame shift/Truncates protein
c.2464dupA	CCA**A**TGG	p.Met822AsnfsX32	10	TM4	3	2.59	Frame shift/Truncates protein
*c.3157dupC	CCC**C**TCA	p.Leu1053ProfsX16	14	ATP loop	1	0.86	Frame shift/Truncates protein
*c.3843dupT	GGT**T**GTG	p.Val1282CysfsX22	18	ATP hinge	1	0.86	Frame shift/Truncates protein
Splice site							
c.1708-5T > G	ttg > tgg	p.IVS4-5 T > G	5	Cu6	3	2.59	abnormal splicing
c.4124 + 5G > A	gag > gaa	p.IVS20 + 5 G > A	20	TM8	1	0.86	abnormal splicing

Novel mutations were denoted by asterisks. Cu,copper binding domain; TM,transmembrane domain.

In the present study, mutations occurred most frequently in exons 8 and 13 (Table 1). No mutations were found in exons 1, 4, 6, 7, 9, or 21. The most frequent WND mutation was p.Arg778Leu, which accounted for 31.9% of the 116 WND alleles studied, followed by p.Pro992Leu (11.2%) and p.Ala874Val (5.17%). Of the 58 unrelated probands, we identified mutations in both alleles (n = 53), in only one allele (n = 3), or in neither allele (n = 2) (Both patients have corneal K-F ring positive, basal urine copper >80 μg/24h and ceruloplasmin < 20 mg/dl). In addition to the 40 mutations, 17 polymorphisms were also identified and are described in detail in Additional file 1 : Supplementary Table S3. Finally, linkage equilibrium was found between the p.Arg778Leu mutation and the p.Leu770Leu polymorphism as has already described [25].

### Mutation spectrum of *ATP7B *in Chinese population

**Figure 5:**
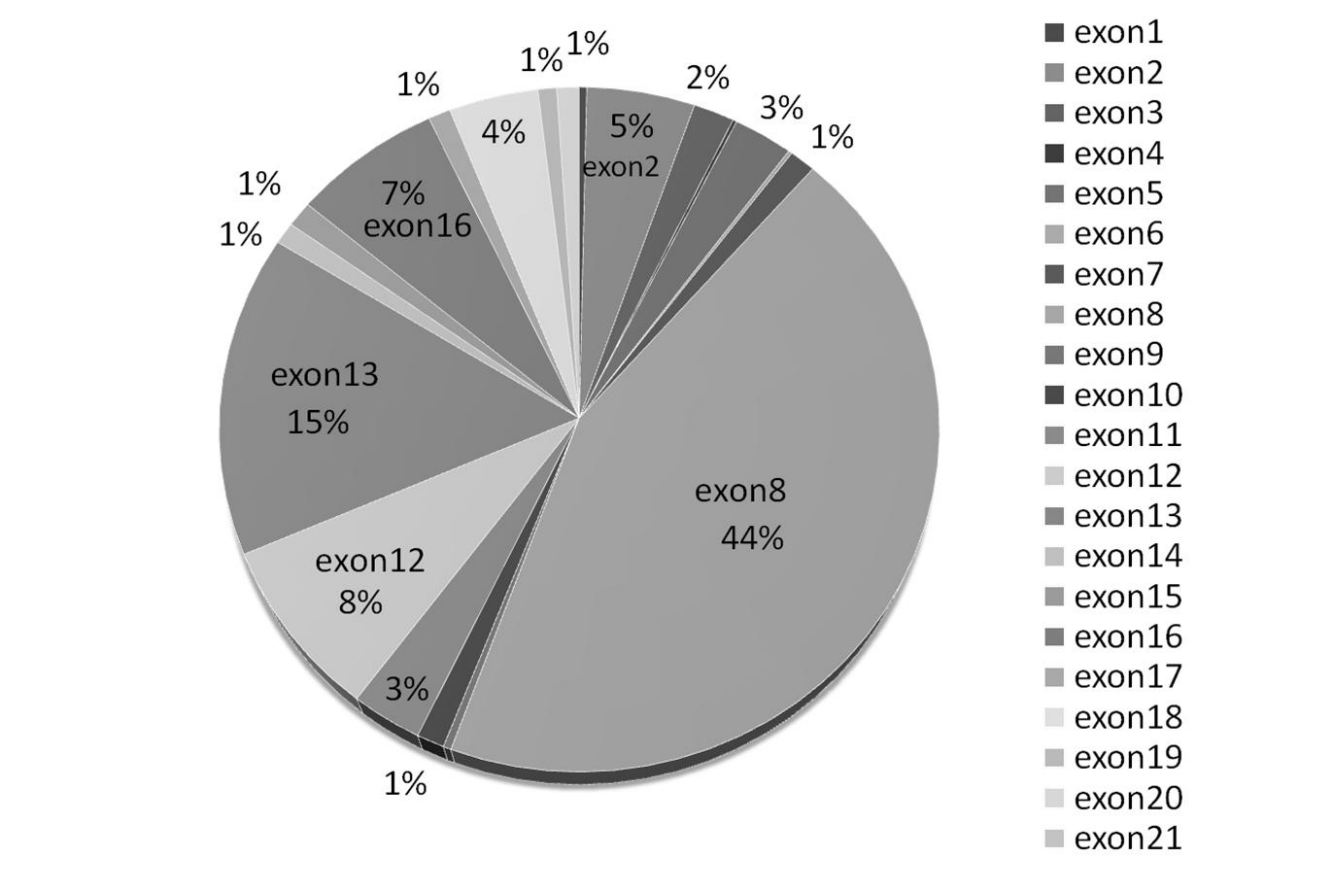
**Distribution and frequency of *ATP7B *gene mutations from 345 patients with Wilson's disease in the Chinese population (including in this study)**.

A PubMed search using the combined search terms of Wilson Disease and mutation(s) retrieved a total of 899 publications. Of these, we analyzed only studies reporting well-defined mutations in Chinese patients [25-31]. To date, a total of 345 Chinese WND patients have been studied (including the present study). Mutations have been detected in all exons except exon 21. Most WND mutations are located in exons 8, 13, 12, and 16, which account for 74.0% of the reported WND alleles (Figure 5). The most frequent WND mutations were p.Arg778Leu and p.Pro992Leu, which account for 50.43% of all the reported WND alleles.

### Genotype-phenotype correlation of the p.Arg778Leu mutation

**Figure 6:**
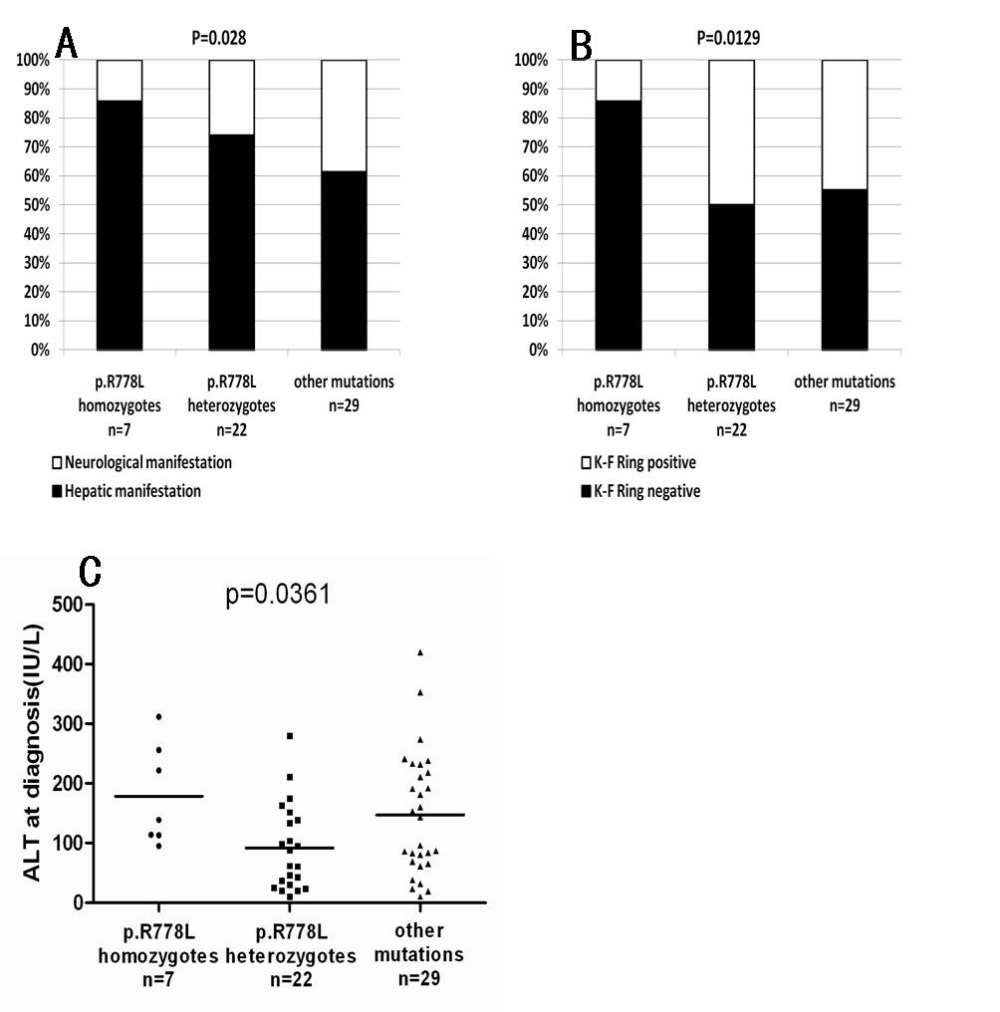
**Correlation of p.Arg778Leu mutation with clinical manifestations (A), cornea Kayser-Fleischer ring rate (B) and ALT levels at diagnosis (C)**.

The 58 WND probands were analyzed for a potential genotype-phenotype correlation with respect to the p.Arg778Leu mutation. We found that patients homozygous for the p.Arg778Leu mutation (n = 7) demonstrated more hepatic manifestations (H1 or H2) and higher serum ALT levels at diagnosis than patients heterozygous for this mutation (n = 22; p = 0.028) or other mutations (n = 29; p = 0.0361) (Figure 6A and 6C). In contrast, only one patient homozygous for the p.Arg778Leu mutation showed neurologic manifestations (N1). K-F rings were also less frequently detected in p.Arg778Leu homozygous patients (14.3%) than in p.Arg778Leu heterozygous patients (50%) or patients with other mutations (44.8%) (p = 0.0129; Figure 6B). No significant differences were observed for age at symptom onset, age at diagnosis, diagnostic delay, serum ceruloplasmin levels, or basal urinary copper levels (Additional file 1 : Supplementary Table S4).

### Nonsense, frameshift, and splice-site mutations

Nonsense, frameshift, and splice-site mutations tend to produce more severe functional impairment than missense mutations. We therefore, subdivided the mutations into two groups: severe mutations (SMs), including nonsense mutations, frameshift mutations and splice site mutations; missense mutations (MMs). According to this classification, we subdivided the patients into three groups: SM/SM, patients possessing two severe mutations (SMs); SM/MM, patients possessing one SM and one missense mutation (MM); MM/MM, patients possessing two missense mutations.

**Figure 7:**
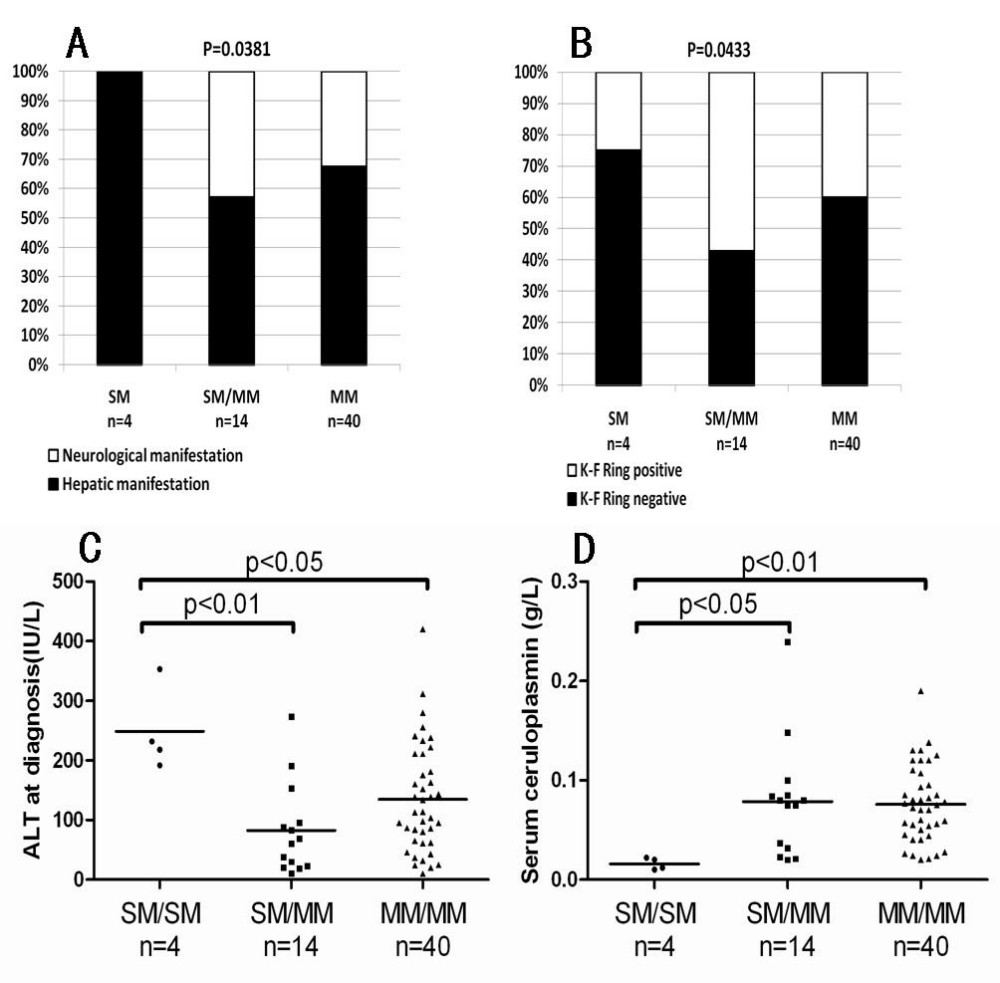
**Correlation of severe mutations (SMs) with clinical manifestations (A), corneal Kayser-Fleischer ring (B), ALT levels at diagnosis (C) and serum ceruloplasmin (D)**. SM, nonsense mutation and frameshift mutations; missense mutations (MM); SM/SM, patients possessing two severe mutations; SM/MM, patients possessing one severe and one missense mutation; MM/MM, patients possessing two missense mutations.

All four patients in the SM/SM group presented with hepatic manifestations (H1 or H2) and one patient presented with acute liver failure; statistics showed significant differences among the three groups (p = 0.0383; Figure 7A). The SM/SM group showed higher ALT levels (p = 0.0061; Figure 7C), lower rate of K-F ring detection (p = 0.0304; Figure 7B), and lower serum ceruloplasmin levels at diagnosis than other groups (p = 0.0065; Figure 7D,). No significant difference was observed for age at symptom onset, diagnostic delay, age at diagnosis, or urinary copper levels (Additional file 1 : Supplementary Table S5).

## Discussion

WND is a potentially fatal disease, but early diagnosis and effective treatment can prevent disease progression in symptomatic patients [9,40] and prevent symptom development in presymptomatic patients. However, clinical manifestations of WND are extraordinarily diverse, and establishing the diagnosis is particularly difficult with children [8,9]. In the present study, children accounted for 80.6% of patients, 27 patients were children younger than 8 years old at diagnosis. The children often lacked typical symptoms: only two of the 27 patients presented with K-F rings, and 60% only had abnormal liver function. Establishment of the diagnosis of WND disease thus poses a major challenge to clinicians. Our data also show that the older the age at diagnosis and the longer the diagnosis delay the likelihood of neurologic manifestations, indicating that early diagnosis and treatment may improve neurologic prognosis. Therefore, rapid and inexpensive diagnostic strategies are urgently needed in China.

Large-scale WND screening of the general population [41] or of specific groups [42] has been considered to be neither feasible nor cost-effective [40]. In 27 patients ( below the age of 8 years old at diagnosis), 96% had abnormal liver function, 93% had low serum ceruloplasmin (<0.2 g/L), 93% had high 24-h urinary copper excretion (> 1 × ULN), 96% lacked neurologic symptoms, and 93% patients lacked K-F rings. We therefore recommend a three-step testing strategy for WND diagnosis for children younger than 8 years old: 1) liver function test (to exclude other causes of hepatitis, such as viral hepatitis etc.); 2) ceruloplasmin or urinary copper test; 3) genetic testing. As physical examinations for nursery school admission have become more common, WND diagnosis in younger children has increased in China (27 of 58 probands were younger than 8 years in the present study). These improvements were gained in part because of genetic testing and the three-step test strategy for WND diagnosis.

Mutations have been detected throughout the *ATP7B *gene, except exon 21, which makes the identification of gene defects particularly challenging [8,9]. Therefore, a rapid and cost-effective strategy is needed for genetic testing. Studies of Chinese patients report that mutations from five exons (8, 12, 13, and 16) account for more than 74% of WND cases [25-31]. Therefore, these four exons should be given high priority for genetic testing in China.

More than 500 mutations have been identified in patients with WND disease [21]. In the present study, we detected 14 novel mutations in the Chinese Han population. Six were nonsense or frameshift mutations; the others were missense mutations that may produce functional defects of transmembrane segments or the ATP-binding domain. We have evaluated the possible impact of missense mutations by using bioinformatic tools SIFT[43], the results showed that all novel eight missense mutations are predicted to affect protein function (data not shown). These results have expanded the knowledge of *ATP7B *mutations and provided valuable information to better understand the function of the ATP7B protein.

The mutation spectrum of *ATP7B *in the Chinese population is quite distinct from Western ethnic populations. Among Chinese patients, the most frequent WND mutations were p.Arg778Leu and p.Pro992Leu, accounting for 50.43% of reported WND alleles. In contrast, the p.His1069Gln, which the most frequent mutation in Western populations [36,44,45], was not detected in any Chinese patients. Clinical features of the p.Arg778Leu mutation differed from those of p.His1069Gln [22,46]. In contrast to p.His1069Gln, patients with homozygous p.Arg778Leu had more hepatic manifestations and fewer neurologic manifestations than patients with heterozygous p.Arg778Leu or other mutations [26,27]. Nonsense and frameshift mutations were more strongly associated with more hepatic manifestations, higher ALT levels, fewer neurologic manifestations, and lower ceruloplasmin levels than other mutations. These findings indicate that the type of mutation can predict clinical manifestations. Interestingly, age at diagnosis, but not genotype, was correlated with urinary copper concentration. This information may account, in part, for the difficulty of WND diagnosis in children [47,48].

This correlation between the p.Arg778Leu mutation and clinical features may be due to functional defects in the ATP7B protein, as demonstrated by the yeast complementation assay [49]. Under iron-limited conditions, p.Arg778Leu mutant constructs were unable to rescue growth defects in *Saccharomyces cerevisiae *lacking the CCC2 gene, but confocal images shows properly localized in COS-7 cell,[50], which suggests that the p.Arg778Leu mutation disrupts the copper channel. In contrast, frameshift/nonsense mutations can produce a premature termination codon, potentially leading to mRNA degradation by the RNA surveillance mechanism [51], or a non-functional truncated protein, which causes severe clinical phenotypes [35].

In summary, evaluating genotype-phenotype correlations in WND will help understand the pathogenesis of WND. The p.Arg778Leu, nonsense, and frameshift mutations potentially lead to more severe disease at an earlier age, usually with hepatic disease manifestations. However, the genotype of *ATP7B *gene may not completely explain the phenotypic variability in WND patients. Other factors that affect disease severity may include levels of copper in diet or other genetic factors [52,53].

We reported the mutation spectrum in a total of 62 Chinese WND patients from 58 unrelated families. Fourteen novel mutations were identified and the relationship between genotypes and phenotypes were analyzed. These results increase knowledge about the population genetics of WND in China. The functional effects of these new mutations require further investigation.

## Conclusions

In conclusion, we identified 14 novel mutations and found that the spectrum of mutations of *ATP7B *in China is quite distinct from that of Western countries. The mutation type plays a role in predicting clinical manifestations. Genetic testing is a valuable tool to detect WND in young children, especially in patients younger than 8 years old. Four exons (8, 12, 13, and 16) and two mutations (p.Arg778Leu, p.Pro992Leu) should be considered high priority for cost-effective testing in China.

## Competing interests

The authors declare that they have no competing interests.

## Authors' contributions

XHL performed the literature review, obtained the clinical data, Statistical analysis and drafted the manuscript, with contributions from YLu, YL, YH, JSW, XXZ. YLu, YL, QCF, JX, GQZ, FZ, QMG, DMY, YH, DHZ, XFK organized the field survey for data collection and obtained the clinical data. XHL, YLu, YL, ZML, XFK, JSW, XXZ were responsible for the design of the study. XHL, YLu, YL were responsible for analysis and interpretation of data. XXZ, JSW were responsible for critical revision of the manuscript for important intellectual content. All authors read and approved the final manuscript.

## Pre-publication history

The pre-publication history for this paper can be accessed here:

http://www.biomedcentral.com/1471-2350/12/6/prepub

## Supplementary Material

Additional file 1 **Supplementary table S1**. Clinical data of 58 WD probands and correlation with clinical manifestation Supplementary table S2 Clinical data of 58 WD probands and correlation with cornea K-F Ring detect Supplementary table S3 ATP7B polymorphisms found in Chinese Wilson disease patients Supplementary table S4 Clinical data of 58 WD probands and correlation with R778L Supplementary table S5 Clinical data of 58 WD probands and correlation with severe mutation Supplementary figure S1. 14 Novel mutations. Arrows indicate single base substitutions; underlining indicates deleted bases; caret character "^" indicates the inserted bases. Click here for file
