# Supplementary material for: Clinical and molecular characterization of Wilson's disease in China: identification of 14 novel mutations
Source: BMC Med Genet. 2011 Jan 11;12:6. doi: 10.1186/1471-2350-12-6 (PMC3025937; doi:10.1186/1471-2350-12-6)

Supplementary table S1 Clinical data of 58 WD probands and correlation with clinical manifestation

	Total	neurological manifestation	Hepatic manifestation	Overall statistical significance
Number (n)	58	19(32.7%)	39(67.3%)	
Gender	23F/35M	7F/12M	16F/23M	p=0.7598(Chi-Square test)
Age at diagnosis (years)	8.38(2.67～61)	13.67(5.5～46)	7.33(2.67～61)	P<0.0001*
Age at symptom onset(years)	7.56(2.5～60.75)	11.75(5～38)	6.27(2.5～60.75)	p=0.0003*
Diagnostic delay (months)	4 (0.25～96)	24(1～96)	3(0.25～60)	p<0.0001*
Kayser–Fleischer ring(n)	25/58(43.1%)	16(84.21%)	9(23.08%)	P<0.0001(Chi-Square test)
ALT at diagnosis(IU/L)	130±94.28	94±72.86	178.7±84.36	p=0.0076(t test)
Serum ceruloplasmin (mg/dl)	7.35(1～23.9)	7.69(2～13.8)	6(1～23.9)	p=0.1905*
24h urinary copper(μg/day)	222.5(18～1727)	336(30～1727)	188(18～991)	p=0.0149*

*This data which were not normally distributed in the analyzed population were presented as median and range and were compared between groups with Mann–Whitney U test.

Supplementary table S2 Clinical data of 58 WD probands and correlation with cornea K-F Ring detect

	Total	K-F Ring positive	K-F Ring negative	Overall statistical significance
Number (n)	58	25(43.1%)	33(56.9%)	
Gender	23F/35M	10F/15M	13F/20M	p=0.9627(Chi-Square test)
Age at diagnosis (years)	8.38(2.67～61)	13(3.3～61)	6.33(2.67～26)	P<0.0001*
Age at symptom onset(years)	7.56(2.5～60.75)	11.67(3.25～60.75)	5.38(2.5～22.67)	P<0.0001*
Diagnostic delay (months)	4 (0.25～96)	6(0.25～96)	3(0.5～72)	p=0.0556*
Neurological manifestation(n)	19(32.7%)	16(64%)	3(9.09%)	p<0.0001(Chi-Square test)
Hepatic manifestation (n)	39(67.3%)	9(36%)	30(90.01%)	p<0.0001(Chi-Square test)
ALT at diagnosis(IU/L)	130±94.28	70.64±62.05	170.5±95.04	p<0.0001(t test)
Serum ceruloplasmin (mg/dl)	7.35(1～23.9)	7.5(3.85～13.8)	7.2(1～23.9)	p=0.5718*
24h urinary copper(μg/day)	222.5(18～1727)	336(83～1727)	130.2(18～601)	p=0.0002*

*This data which were not normally distributed in the analyzed population were presented as median and range and were compared between groups with Mann–Whitney *U* test.

Supplementary table S3 ATP7B polymorphisms found in Chinese Wilson disease patients

Nucleotide change	Codon change	Amino acid change	Exon/intron	Number of variant alleles	SNP no
-520 C > T	5'UTR	5'UTR	1	23	rs9563084
c.-129_-125del	5'UTR	5'UTR	1	31	rs28362531
-75 A > C	5'UTR	5'UTR	1	55	rs2277448
c.1216 T > G	TCT > GCT	p.Ser406Ala	2	57	rs1801243
c.1366G > C	GTG > CTG	p.Val456Leu	3	39	rs1801244
2144-53 T > C	IVS3-53 T > C	intronic	intron3	33	rs2147363
c.2310 C > G	CTC > CTG	p.Leu770Leu	8	36	
c.2448-25 G > A	IVS9-25 G > A	intronic	intron9	8	rs9526811
c.2495A > G	AAG > AGG	p.Lys832Arg	10	42	rs1061472
c.2576-30 G > A	IVS10-30 G > A	intronic	intron10	30	rs2281814
c.2855 G > A	AGA > AAA	p.Arg952Lys	12	30	rs732774
c.2866-13 G > C	IVS12-13 G > C	intronic	intron12	2	rs7325983
c.3318 C > T	GTC > GTT	Val1106Val	15	1	
c.3419 C > T	GCC > GTC	p.Ala1140Val	16	65	rs1801249
c.3444 T > A	ATT > ATA	p.Ile1148Ile	16	2	
c.3630 G > A	CAG > CAA	p.Gln1210Gln	17	1	
3903+6 T > C	IVS18+6G > A	intronic	intron18	27	rs2282057

Supplementary table S4 Clinical data of 58 WD probands and correlation with R778L

	Total	R778L homozygotes	R778L heterozygotes	Other mutations	Overall statistical significance
Number (n)	58	7(12.1%)	22(37.9%)	29(50%)	
Gender	23F/35M	3F/4M	8F/14M	12F/16M	p=0.066(Fisher's test)
Age at diagnosis (years)	8.38(2.67～61)	6.42(4.67～11.67)	8.13(3.16～61)	9.5(2.67～46)	p=0.2038*
Age at symptom onset(years)	7.56(2.5～60.75)	4.92(3.67～10.34)	8.07(2.99～60.75)	7.59(3.5～38)	p=0.3431*
Diagnostic delay (months)	4 (0.25～96)	4(1～24)	3(0.25～72)	6(0.5～96)	p=0.6468*
Neurological manifestation(n)	19(32.7%)	1(14.28%)	6(27.27%)	12(41.38%)	P=0.0286(Fisher's test)
Hepatic manifestation (n)	39(67.3%)	6(85.72%)	16(72.73%)	17(58.62%)	P=0.0286(Fisher's test)
Kayser–Fleischer ring(n)	25/58(43.1%)	1(14.29%)	11(50%)	13(44.83%)	P=0.0129(Fisher's test)
ALT at diagnosis(IU/L)	130±94.28	178.7±84.36	91.67±71.93	147.3±103.1	p=0.0361(ANOVA)
Serum ceruloplasmin (mg/dl)	7.35(1～23.9)	5.5(2.1～8)	6.45(2～13)	8.2(1～23.9)	p=0.1091*
24h urinary copper(μg/day)	222.5(18～1727)	240(80～521.5)	225(18～991)	191(40～1727)	p=0.7966*

*This data which were not normally distributed in the analyzed population were presented as median and range and were compared between groups with Kruskal–Wallis ANOVA test.

Supplementary table S5 Clinical data of 58 WD probands and correlation with severe mutation

	Total	SM	SM/MM	MM	Overall statistical significance
Number (n)	58	4(6.89%)	14(24.14%)	40(68.97%)	
Gender	23F/35M	2F/2M	6F/8M	15F/25M	p=0.0824(Fisher's test)
Age at diagnosis (years)	8.38(2.67～61)	7.63(6.33～9.5)	10.5(3～13.67)	7.96(2.67～61)	p=0.8620*
Age at symptom onset(years)	7.56(2.5～60.75)	7.35(6.27～9.46)	6.7(2.96～12.97)	7.71(2.5～60.75)	p=0.6987*
Diagnostic delay (months)	4 (0.25～96)	0.58(0.5～6)	6.5(0.5～96)	4.5(0.25～96)	p=0.1228*
Neurological manifestation(n)	19(32.7%)	0(0%)	6(42.86%)	13(32.5%)	P=0.0381(Fisher's test)
Hepatic manifestation (n)	39(67.3%)	4(100%)	8(57.14%)	27(67.5%)	P=0.0381(Fisher's test)
Kayser–Fleischer ring(n)	25/58(43.1%)	1(25%)	8(57.14%)	16(40%)	P=0.0433(Fisher's test)
ALT at diagnosis(IU/L)	130±94.28	225(192～353)§	82.5±76.33	134.8±91.52	P=0.0061*
Serum ceruloplasmin (mg/dl)	7.35(1～23.9)	1.6(1～2.2)	7.75(2～23.9)	7.35(2～19)	P=0.0065*
24h urinary copper(μg/day)	222.5(18～1727)	245(147～601)	123(40～788)	237(18～1727)	p=0.5972*

SM, Nonsense mutation and frameshift mutations; MM,missense mutations; SM/SM, patients possessing two 'severe' mutations; SM/MM, patients possessing one SM and one missense mutation; MM/MM, patients possessing two missense mutation.

*This data which were not normally distributed in the analyzed population were presented as median and range and were compared between groups with Kruskal–Wallis ANOVA test.

§"sample size too small" can not check the normality and were presented as median and range.

Supplementary figure S1 Novel mutations. Arrows indicate single base substitutions; underlining indicates deleted bases; caret character “^” indicates the inserted bases.

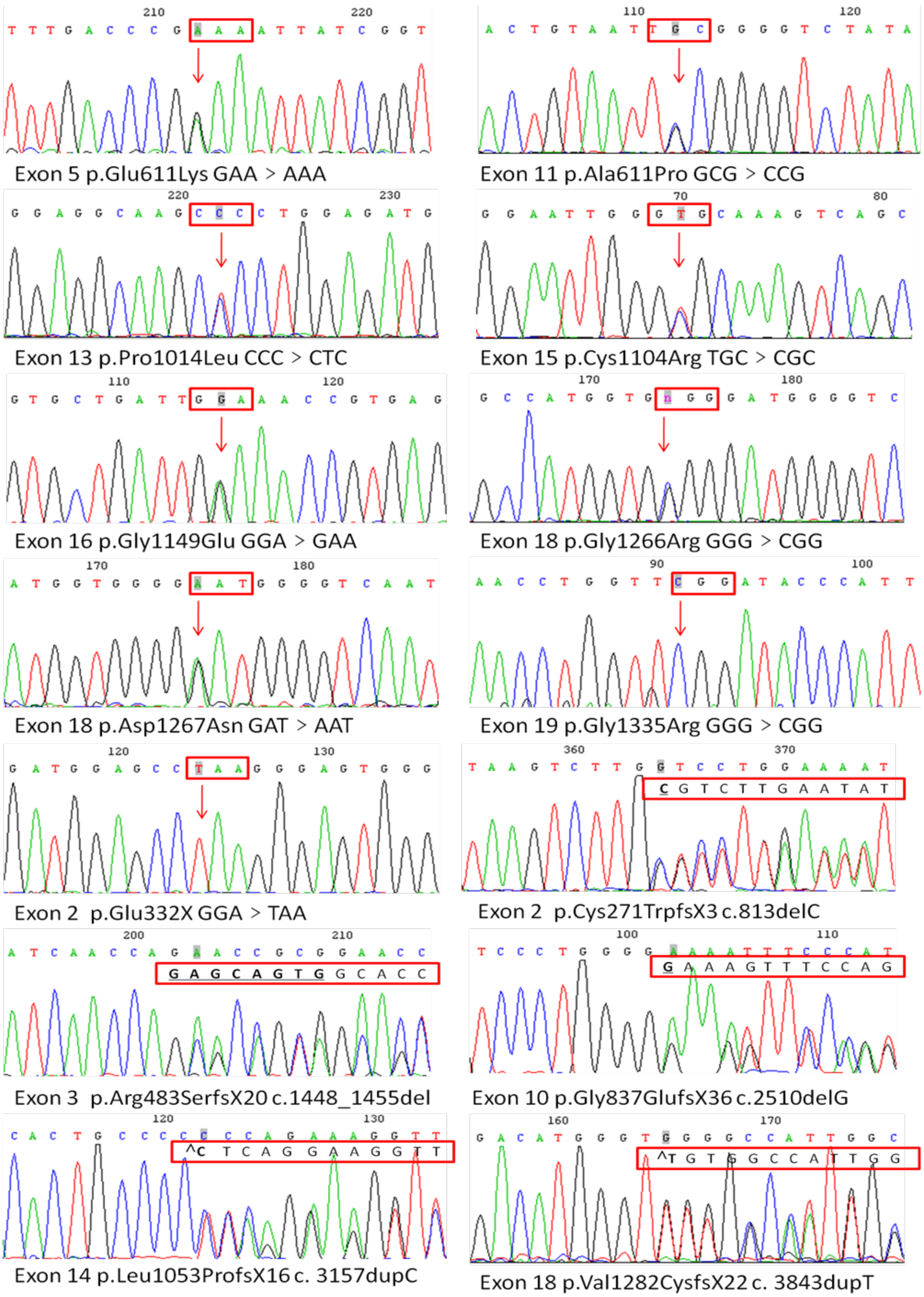

Supplement: Additional file 1 — Supplementary table S1. Clinical data of 58 WD probands and correlation with clinical manifestation Supplementary table S2 Clinical data of 58 WD probands and correlation with cornea K-F Ring detect Supplementary table S3 ATP7B polymorphisms found in Chinese Wilson disease patients Supplementary table S4 Clinical data of 58 WD probands and correlation with R778L Supplementary table S5 Clinical data of 58 WD probands and correlation with severe mutation Supplementary figure S1. 14 Novel mutations. Arrows indicate single base substitutions; underlining indicates deleted bases; caret character "^" indicates the inserted bases. [file 1471-2350-12-6-S1.PDF]
